# Cold Dash in Congestive Intermittents

**Published:** 1837

**Authors:** 


*February*, 1837.

---

### Art. VII.—The Cold Dash in Congestive Intermittents.

*Cases illustrating the efficacy of the Cold Affusion in the Chill of Congestive Intermittent Fever.* Extracted from the Inaugural Thesis of Achilles Whitlocke, M. D., of North Alabama; defended in the Cincinnati College, February, 1837.

There is a disease, which often pervades the valley of the Tennessee river, in North Alabama, which has been described as an algid intermittent, but is known to physicians by the common appellation of *congestive fever*. Its attack is quite irregular and insidious; commencing at the extremities, it finally steals over the whole body, and reduces its temperature much below the natural standard. It is characterised by a protracted cold stage. The system partially recovers its warmth before the recurrence of the chill; but the hot stage, which is so often looked for, is never developed; in its stead, we have the stage of oppression. The patient complains of great internal heat, inordinate thirst, a sense of weight about the epigastrium, great restlessness, difficult and confined respiration, contracted countenance, skin preternaturally cold, pulse quick and feeble, varying from 100 to 120

strokes in a minute. In this stage of suffocated excitement, there is a lesion of secretion throughout the alimentary system, which cannot be repaired until the oppression is removed. The common mode of treatment in this disease, has, heretofore, been almost confined to the warm bath, sinapisms, epispastics, etc., with such cathartic medicines as have a special influence on the liver. This mode is often successful, yet I have seen cases, which have been prolonged by the practice; and many, which seemed to be beyond the reach of such measures, yielded readily to the affusion of cold water on the naked body. The manner of application consists simply, in pouring fifteen or twenty gallons of cold water on the bare body, after which, the patient is to be wiped dry, returned to his bed and covered thickly with clothes.

Reaction, which has already commenced, now gradually increases, the pulse is slow, full and soft, respiration is easy and the patient derives so much relief, as to fall into a state of quietude and refreshing sleep, which often continues for several hours. At the expiration of a few hours, the general reaction is established, and nothing more is required, to arrest all morbid symptoms, than the liberal use of quinine and mild laxatives: such as blue mass, rhubarb and oil. He, who never witnessed the effects of cold water in this fatal disease, would be as much surprised as gratified. It appears so absolutely paradoxical, that cold water should restore warmth to a surface preternaturally cold, that the strongest prejudices are always to be encountered against its administration. I confess that I myself, prior to witnessing its administration, held its use in great distrust; but being forced to its application as a dernier resort, and observing, contrary to my expectations, its benign, remedial influence, I was compelled to acknowledge its powerful agency, and my subsequent experience has fully confirmed my confidence in its efficacy.

Case 1. The first patient on whom I employed it, was an athletic black man, 22 years of age, who had enjoyed uninterrupted health through life. I saw him on the third day after his attack; and prior to my visit, he had taken two doses of calomel and oil, which were, at this time, bringing off watery evacuations. On examination, I found him very restless, with a slow feeble pulse, which could scarcely be observed; his respiration difficult, his eyes half closed, and his whole body preternaturally cold. He was aroused, so as to answer, indistinctly, a few questions, and would then fall into an apparent sleep, which would only last a few minutes, before he would change his position. I commenced stimulating him with opium and camphor; and directed a warm bath to be prepared immediately, in which he was placed and retained about fifteen minutes, without any sensible change in his pulse. He was wiped and returned to bed, sinapisms were then applied to the epigastrium and extremities, and internal stimulants were given *per anum et per ore*, for five hours without any important change. These means having proved so ineffectual, I determined to resort forthwith to the experiment of the cold affusion. The patient being stripped and placed on the floor, I poured on his body, from an elevation of two feet, about twenty gallons of water, made colder by the addition of common salt, after which he was wiped and put to bed. At the expiration of ten minutes, evident symptoms of reaction had commenced; his pulse rose, and his face which had exhibited a shrunken appearance, now became enlivened, and the temperature of his body was much increased. But this reaction did not continue long, for in an hour and a half, all the distressing symptoms again appeared, and as no time was to be lost, I repeated the affusion as before. In a few minutes, the patient was so sensible of his altered condition, as to express it himself; and in a short time fell into a sleep which lasted several hours, after which, general reaction was present, and the temperature of his body rose to near its natural standard. I administered quinine and gentle laxatives for several days afterwards, which restored the patient to his former health and occupation in about two weeks. Since that time, I have employed it, in all stages of congestive fever, and it has been almost uniformly attended with the same signal success. I shall presently relate other cases in which the patients have been, as it were, snatched from the jaws of death, by this powerful remedy.

To explain its *modus operandi*, is a task, which I dare not undertake, yet I believe its action is simultaneous, on both the nervous and vascular systems, and through them on the secretory organs; for it is not uncommon during the affusion, to observe a copious flow of urine from the bladder, which may be always considered as strongly indicative of a speedy improvement. The common practice in this region, is to repeat the affusion, according to circumstances, until general reaction is brought on, which it seldom fails to produce; though like all other remedies, it sometimes falls short of our more sanguine expectations.

The administration of this agent in the collapse of fever, so far as I am informed, originated with Dr. Thomas Fearn, of Huntsville, Alabama; whose reputation both as a physician and surgeon, is too well known to the profession in the south to need my humble testimony. Living in a region of country, where the diseases are generally violent, he resolved, as a dernier resort, on the experiment of cold water, in the stage of collapse, of the disease now under consideration, and his experiment was not fruitless, for in numerous instances, he and his enlightened colleague, Dr. Erskine, have employed it with unprecedented success; and they do not hesitate to recommend it to the profession, as an agent of superior efficacy to any other they have ever employed. They farther believe, that, where the susceptibility of impression is not entirely destroyed, and where no vital organ has sustained an irrecoverable injury, the affusion of cold water, will in almost every case, be attended with complete success. To exemplify its effects fully in this malady, I will here detail a few additional cases, which came under my own care and observation, within the last three years.

Case 2. On the 3d September, 1834, I was called to see a black man, the property of Mr. F., aged 34 years, and of good constitution. I found him very restless, with a small, quick pulse of one hundred and thirty-five beats to the minute, and bathed in a cold clammy sweat over its whole surface; he complained of great weight in or about the epigastrium, had an insatiate thirst for cold drinks only; his respiration was difficult, and his physiognomy was shrunken. I learned from the overseer, that, he had had a chill two days previous, and one on the morning of the present day, which was now nearly night, and had become much worse since the approach of the sweating stage. Fully understanding the case, as I thought, I ordered some cold well-water to be brought, and immediately poured on his naked body about twenty gallons, having finished, the patient was so much relieved as to return to bed without assistance. In a short time, his oppression was removed, the heat of the surface returned, and he fell into a refreshing sleep. His pulse gradually rose, and became open, full, and less frequent, his respiration easy, and general reaction was present when he awoke. Nothing but the free use of quinine and mild laxatives was afterwards necessary to restore him to his former health.

Case 3. September 12th, 1834, I was called to see Mr. D., a man of intemperate habits, aged forty-six years. His employment was very irregular, consisting of fishing and hunting for a sustenance. I found him as usual in these cases, covered with a cold, clammy sweat, turning from side to side, with hurried and difficult respiration, and complaining of a feeling, as if a cord were tied around his chest, tongue moist and a little white, with a pale and shrunk countenance, a pulse more frequent and small than any I ever recollect to have felt before or since. I did not hesitate to give him the cold douche immediately, and in a very short time his condition was much ameliorated. Reaction commenced as usual, but did not continue long, before the unpleasant symptoms returned, which induced me to repeat again the cold affusion. The next application was made with water, cooled by the addition of salt; after having poured fifteen gallons of this on the body, the patient was wiped dry, and placed between two blankets, on a feather bed and well covered. I soon discovered that his countenance and respiration, indicated a propitious change, and in a few minutes he was disposed to sleep. His pulse had now become slow, free and open, the general warmth of the system was reproduced he rested well during the remainder of the night. On the next morning, he commenced the use of quinine, in doses of twenty grains every hour, until his head was affected, which prevented the return of another paroxysm and he soon recovered.

Case 4. September 20th, 1836, the daughter of Mr. B. aged eight years, was taken on Monday morning, with a chill and the usual symptoms, or at least nothing appeared uncommon in this or the paroxysm, which occurred on Wednesday succeeding. During the week she had taken one or two doses of calomel and castor oil, and in the intermission, when the pulse justified it, quinine was also administered. Early on Saturday morning, she had another chill, which like those that preceded it, exhibited nothing indicative of danger or calculated to alarm us, but the excitement upon reaction was excessive. A hot dry skin, insatiable thirst, a full resisting pulse, suffused countenance, violent throbbing of the carotid and temporal arteries, and, finally, every distressing symptom were present. I directed warm diluent drinks, and leaving her did not return until 9 o'clock, P. M. When I returned, I found her covered entirely with a cold clammy sweat, pulse less and speechless; her flesh cold as marble, her countenance was so much changed and shrunk since I left her, that I would have scarcely recognised her at any other place. I learned from the father, that her pulse began to sink in a short time after my departure; when she made great complaint, and continued her complaining as long as any pulse could be discovered at the wrist. Her symptoms during this stage, were precisely the same as generally characterise this disease—very great oppression and restlessness, which the patient cannot control. Having entire confidence in the sudden and powerfully stimulant effects of cold water, I administered it without hesitation. Nor was my confidence unfounded, for immediately after the first application, much more warmth was perceived, though little arterial action was to be felt except in the carotid arteries. After the lapse of an hour and a half, I repeated the affusion, and the patient was returned to bed, and well covered. The warmth of the whole system now gradually increased, the pulse became regular and soft, and she continued to improve during the remainder of the night. The little sufferer after the second affusion, fell into a sleep, which continued pretty much throughout the night. By morning, she was to appearance well, being anxious to get up and indulge in eating. Her pulse now being regular and natural, I administered quinine liberally, for several days; which, together with mild laxatives; soon restored her to usual health and vigor.

In detailing these few cases, in which I have endeavored to present all the minutiæ of morbid phenomena which generally prevail in the cases refered to, and which seem to yield almost exclusively to the administration of cold water, the effects produced by such administration, are I trust sufficiently plain and numerous to justify all I have said. That it is a therapeutic agent of great efficacy, I am sure; and I believe it entitled to the confidence and consideration of the whole profession, in the treatment of the forms of disease now under consideration. I have often witnessed its salutary effects, when all other means had proved abortive; especially in those cases where the internal organs are overloaded and forced to retain and circulate blood, that belongs to the exterior vessels. In this state of lesion and oppression, occasioned by an unnatural accumulation of fluids, I have seen it, by virtue of its own specific action, restore secretion, and diffuse heat and the circulation to the exterior, so speedily, as to enable the patient to arise out of the state of lethargy and prostration, and walk from the bathing tub to the bed without assistance. I have often given calomel in congestive conditions of the system, with the effect, as I believe, of augmenting the general irritation, it being entirely impotent as a cathartic. I firmly believe, that in all stages of extreme congestion, it has no power over the secretions; but that the profuse quantities, so often given by southern practitioners, have a deleterious tendency, and increase the sufferings of the patient. Any agent then, that can arouse the energies of the system, and restore activity to the torpid and engorged organs, must necessarily be a valuable remedy—and such I regard the Cold Affusion.

